# Effects of Anodal Left DLPFC tDCS on Panic Severity in Panic Disorder Patients With and Without Comorbid Rhinosinusitis and Nasal Septum Deviation

**DOI:** 10.1002/brb3.71508

**Published:** 2026-05-24

**Authors:** Roghayeh Mohammadi, Ahmad Alipour, Zahra Alipour

**Affiliations:** ^1^ Department of Psychology Payame Noor University Tehran Iran; ^2^ Department of Psychology Roudehen Campus, Islamic Azad University Roudehen Iran

**Keywords:** dorsolateral prefrontal cortex, nasal septum deviation, panic disorder, rhinosinusitis, transcranial direct current stimulation

## Abstract

**Purpose**: Panic disorder (PD) is characterized by recurrent, unexpected panic attacks and impaired top‐down modulation of the fear circuitry, notably left dorsolateral prefrontal cortex (DLPFC) hypoactivity and dysregulation of respiratory‐panic pathways. Transcranial direct current stimulation (tDCS) is a non‐invasive brain stimulation technique that modulates cortical excitability by delivering low‐intensity electrical currents through scalp electrodes. Chronic rhinosinusitis (CRS) and nasal septum deviation (NSD) commonly disrupt nasal breathing and sleep while increasing anxiety vulnerability. This study evaluated the efficacy and long‐term stability of anodal tDCS over the left DLPFC in reducing panic severity in PD patients with versus without comorbid CRS or NSD.

**Methods**: Sixty‐six adults with DSM‐5 PD were purposively allocated to PD‐only (*n* = 27), PD + CRS (*n* = 26), and PD + NSD (*n* = 13) groups. All received 25 sessions of anodal tDCS (2 mA, 20 min; anode F3, cathode FP2). Panic severity was assessed with the Panic Disorder Severity Scale (PDSS) at baseline, post‐treatment, and 6‐ and 12‐month follow‐ups. The data were analyzed using mixed ANOVA with Bonferroni‐corrected post‐hoc tests.

**Finding**: A significant time × group interaction was observed (*F* = 7.65, *p* < 0.001, *η_p_* ^2^ = 0.20). All groups showed large, immediate reductions in PDSS scores (57%–65%, *p* < 0.001). Treatment gains were fully maintained at 12 months in the PD‐only and PD + CRS groups, whereas the PD + NSD group exhibited statistically and clinically significant partial relapse by 12 months (*p* = 0.022 versus both other groups).

**Conclusion**: Anodal left‐DLPFC tDCS is highly effective and generally durable for panic disorder. However, untreated nasal septum deviation substantially compromises long‐term stability, probably via persistent interoceptive stress and respiratory dysregulation. Systematic screening and management of upper‐airway patency are recommended to optimize sustained outcomes.

## Graphical abstract

Anodal left‐DLPFC tDCS produces large, rapid remission in panic disorder that is durable at 1 year unless untreated nasal septum deviation is present, which drives partial relapse via persistent respiratory dysregulation.

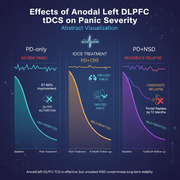

## 1 Introduction

Panic disorder (PD) is a debilitating psychiatric condition characterized by recurrent, unexpected panic attacks (Aljadani et al. 2024). From an evolutionary standpoint, PD represents a dysfunction in brain circuitry in which higher order cortical regions fail to properly inhibit phylogenetically older subcortical structures, particularly the periaqueductal gray (PAG), which orchestrates immediate fight‐or‐flight responses (Shafiee et al. 2024). This functional disconnection between the PAG and cortico‐amygdalar regulatory networks leads to exaggerated autonomic and somatic reactions to benign or neutral stimuli—the hallmark of panic attacks (Guan and Cao 2024). A large body of research has further established a fundamental link between respiratory physiology and panicogenesis. Central CO_2_/H^+^‐chemosensitive regions extend beyond the brainstem to include hypothalamic neurons directly implicated in PD pathophysiology (Eugenín and Richerson 2024). Clinically, patients with PD demonstrate markedly lower CO_2_ thresholds for triggering hyperventilation, intense fear, and escape behavior compared with healthy individuals, highlighting respiratory dysregulation as a core mechanism in the expression of panic (Kyriakoulis and Kyrios 2023).

The integrity of the upper airway is highly relevant in this context, given the high population prevalence of chronic rhinosinusitis (CRS) and nasal septum deviation (NSD) (Shulkin et al. 2025). CRS is characterized by persistent sinonasal mucosal inflammation and increased release of proinflammatory cytokines such as IL‐1β, TNF‐α, and TGF‐β (Li et al. 2024). This chronic inflammatory milieu disrupts sleep quality, in part through elevated TGF‐β and IL‐4, affecting 60%–75% of CRS patients and contributing to elevated rates of comorbid depression (∼25%) and anxiety (∼29%) (Mahdavinia et al. 2017; Rădeanu et al. 2025). Similarly, NSD causes chronic nasal obstruction, reduced quality of life, and significantly higher levels of psychological distress, including more frequent and severe anxiety and depressive symptoms (Lee et al. 2021; Akkoca et al. 2020).

Transcranial direct current stimulation (tDCS) is a safe, non‐invasive brain stimulation technique that modulates cortical excitability and promotes neuroplasticity by delivering low‐intensity electrical current through scalp electrodes. Panic symptom severity—encompassing attack frequency, intensity, anticipatory anxiety, avoidance, and functional impairment—serves as the key dependent variable (Tunnell et al. 2024). The false suffocation alarm (FSA) theory proposes that PD arises from a hypersensitive, lowered threshold of an evolved internal suffocation monitoring system (Preter and Klein 2008). Minor physiological fluctuations misinterpreted as imminent asphyxia activate an extended network involving medullary respiratory centers, hypothalamus, limbic structures, and prefrontal cortex (Leibold et al. 2015). Patients with PD consistently show respiratory instability, pathological CO_2_ hypersensitivity, and increased comorbidity with respiratory disorders such as asthma and Chronic obstructive pulmonary disease (COPD) (Tunnell et al. 2021).

Non‐invasive brain stimulation techniques, particularly tDCS, have emerged as promising non‐pharmacological treatments for anxiety‐spectrum disorders, including PD (Cirillo et al. 2019). Anxiety disorders, including PD, are consistently associated with prefrontal asymmetry and left dorsolateral prefrontal cortex (L‐DLPFC) hypoactivity (Kenwood et al. 2022). Anodal tDCS over the L‐DLPFC (anode at F3 according to the 10–20 EEG system) increases excitability in this critical regulatory hub, thereby enhancing top‐down inhibitory control over hyperactive limbic circuits, particularly the amygdala (Palmisano et al. 2021; Liu et al. 2023). This protocol has repeatedly shown efficacy in alleviating anxiety symptoms across affective disorders (Nishida et al. 2025; Hausman et al. 2024).

While tDCS has been established as a potent treatment for PD, a crucial gap remains in our understanding of how chronic peripheral physical conditions, particularly those that compromise normal respiration—such as CRS and NSD—influence the long‐term stability of the treatment's centrally mediated effects. The present study therefore systematically investigated the efficacy and long‐term durability of 25 sessions of anodal L‐DLPFC tDCS on panic severity in three groups: patients with PD without upper airway comorbidity, patients with PD and comorbid chronic rhinosinusitis (PD + CRS), and patients with PD and comorbid nasal septum deviation (PD + NSD).

## 2 Methods

The current study utilized a three‐group, mixed quasi‐experimental design employing a pretest‐posttest format with two follow‐up assessments. In this design, the factor “Group” (with three levels: PD‐only, PD + CRS, and PD + NSD) served as the between‐subjects variable, and the factor “Time” (with four levels: Pretest, Posttest, 6‐month Follow‐up, and 12‐month Follow‐up) served as the within‐subjects variable. The statistical population comprised all individuals diagnosed with PD who sought treatment at specialized psychological and counseling centers between Summer 2024 and Summer 2025. A total of 66 participants were selected from this population using purposive sampling and were subsequently confirmed by a certified psychiatrist according to *DSM‐5* diagnostic criteria. They were allocated to three distinct groups: Group 1: PD patients without any comorbid CRS or NSD (*n* = 27); Group 2: PD patients concurrently diagnosed with CRS (*n* = 26); and Group 3: PD patients concurrently diagnosed with NSD (*n* = 13).

Rigorous inclusion and exclusion criteria were applied to ensure result validity. Inclusion criteria mandated: age between 18 and 55 years, a primary diagnosis of PD (with or without agoraphobia), and scoring above the clinical cutoff on the panic severity scale. Exclusion criteria included history of seizure disorders, presence of metallic implants in the head, pregnancy, concurrent diagnosis of psychotic disorders (e.g., schizophrenia) or bipolar disorder, active substance abuse, or the initiation or dose change of any new psychological or pharmacological treatment during the intervention period. The study was approved prospectively by the Ethical Committee of Payame Noor University. All participants provided written informed consent, and the study received full ethical approval from the Institutional Research Ethics Committee of the University. Participants were guaranteed confidentiality of their data and the right to withdraw from the study at any time without penalty.

### 2.1 Measure

The Panic Disorder Severity Scale (PDSS) is a clinician‐administered, seven‐item instrument widely regarded as the gold‐standard tool for assessing panic disorder severity. It measures five core dimensions over the past month (or past week for repeated assessments): frequency of full and limited‐symptom panic attacks, distress during attacks, anticipatory anxiety, agoraphobic fear and avoidance, interoceptive fear and avoidance, impairment in work/school functioning, and impairment in social/family functioning. Each item is scored on a 0–4 Likert scale (0 = none/absent, 4 = extreme/very severe), producing a total score ranging from 0 to 28. Clinically significant panic disorder is indicated by scores ≥8; a ≥40% reduction from baseline is considered treatment response, and scores ≤5 typically denote remission (Shear et al. 1997). The PDSS exhibits excellent psychometric properties, including high internal consistency (Cronbach's *α* = 0.88–0.93), strong test‐retest reliability, robust convergent and discriminant validity, and superior sensitivity to clinical change in both pharmacological and psychological interventions (Shear et al. 2001; Wuyek et al. 2011). In the current sample, internal consistency at pretest was *α* = 0.91.

### 2.2 Procedure

After initial screening and confirmation of a *DSM‐5* diagnosis of panic disorder by a board‐certified psychiatrist, all participants provided written informed consent. Baseline panic severity was assessed using the PDSS. Subsequently, participants received 25 daily sessions (5 days/week) of anodal tDCS (2 mA, 20 min; anode over F3—left dorsolateral prefrontal cortex; cathode over FP2—right orbitofrontal cortex) at a specialized neuropsychology clinic under the direct supervision of a trained clinical neuroscientist. No new psychotropic medication or psychotherapy was initiated or dose‐adjusted during the active treatment phase. The PDSS was readministered immediately after the last tDCS session (posttest) and at 6‐month and 12‐month follow‐ups. This repeated‐measures design permitted the evaluation of both acute treatment response and long‐term maintenance of therapeutic gains across the three groups (PD‐only, PD + CRS, and PD + NSD).

### 2.3 tDCS Protocol

All three groups received an identical anodal tDCS protocol targeting the L‐DLPFC. Stimulation was delivered using a CE‐certified direct current stimulator with two saline‐soaked surface sponge electrodes (5 × 7 cm). The anode was placed over F3 and the cathode over FP2, according to the international 10–20 EEG system. Current intensity was set at 2.0 mA and delivered for 20 min per session, with 30‐s ramp‐up and ramp‐down periods. Participants underwent 25 sessions over 5 consecutive weeks (five sessions per week, Monday to Friday). This montage and these parameters were chosen to increase cortical excitability in the L‐DLPFC, thereby enhancing top‐down regulation of hyperactive limbic and subcortical fear/panic circuits. Impedance was kept below 10 kΩ throughout each session, and adverse effects were systematically monitored and recorded.

### 2.4 Management of Comorbid Conditions

With regard to the management of comorbid conditions, participants in the PD + CRS group had received standard medical treatment for chronic rhinosinusitis (including nasal corticosteroids and, where clinically indicated, short courses of antibiotics) as prescribed by their otolaryngologists prior to study enrollment. No surgical intervention (e.g., functional endoscopic sinus surgery) was performed in this group during the study period. In the PD + NSD group, all participants had radiologically confirmed nasal septum deviation but had not undergone septoplasty or any other surgical correction before or during the study. No new medical treatments or surgical interventions for CRS or NSD were initiated during the tDCS treatment phase or the follow‐up period. This information was verified through detailed medical history review, participant self‐report, and confirmation with the participants’ treating physicians where available.

### 2.5 Data Analysis

Data were analyzed using IBM SPSS Statistics Version 26. To examine changes in panic severity (PDSS total score) across the four assessment points and between the three groups, a mixed between‐within subjects analysis of variance was conducted, with Group (PD‐only, PD + CRS, PD + NSD) as the between‐subjects factor and Time (pretest, posttest, 6‐month follow‐up, 12‐month follow‐up) as the within‐subjects factor. Greenhouse–Geisser correction was applied when the assumption of sphericity was violated. Significant effects were followed by Bonferroni‐corrected post hoc pairwise comparisons. The alpha level was set at *p* < 0.05 (two‐tailed). Effect sizes are reported as partial eta‐squared (*η_p_* ^2^).

## 3 Results

The study included 66 patients with panic disorder (47 females [71.2%], 19 males [28.8%]) who completed all assessments, with a mean age of 44.8 years (SD = 6.9; range, 24–55 years). The groups were comparable in terms of sex distribution (*χ* ^2^ = 5.25, *p* = 0.074) and educational attainment (*F* = 1.42, *p* = 0.249). A modest but significant age difference emerged (*F* = 4.85, *p* = 0.011, *η_p_* ^2^ = 0.13); post hoc Bonferroni tests indicated that the PD + NSD group was younger (*M* = 40.7 years, SD = 6.6) than the PD + CRS group (*M* = 47.6 years, SD = 6.8; *p* = 0.009), while the PD‐only group (*M* = 43.1 years, SD = 7.6) did not differ significantly from either (*p* > 0.05). Baseline PDSS scores were equivalent across the groups (*F* = 0.07, *p* = 0.931), ensuring no pretreatment disparities in panic severity. No adverse events related to tDCS were reported, and adherence to the 25‐session protocol was 100%.

Descriptive statistics for PDSS total scores (range, 0–28) at each assessment point are summarized in Table 1. All groups entered treatment with clinically severe panic (mean scores >20, exceeding the ≥8 cutoff for significant impairment). Post‐treatment, mean scores dropped substantially (11.9‐ to 13.8‐point reductions, representing 57%–65% improvement), entering the mild range (≤12). The PD‐only and PD + CRS groups sustained these gains through 12 months, with scores remaining below the remission threshold (≤5–8). In contrast, the PD + NSD group showed a progressive symptom uptick after the 6‐month mark, rising to moderate severity (15.15) by 12 months—still improved from baseline but indicating partial relapse.

**TABLE 1:** Means and standard deviations of PDSS total scores by group and assessment phase.

Assessment	PD‐only (*n* = 27), mean ± SD	PD + CRS (*n* = 26), mean ± SD	PD + NSD (*n* = 13), mean ± SD	Total (*N* = 66), mean ± SD
Pretest	20.70 ± 6.48	20.92 ± 6.04	21.38 ± 6.61	20.92 ± 6.31
Posttest	7.89 ± 4.21	8.42 ± 4.21	12.23 ± 5.68	9.05 ± 4.92
6‐month follow‐up	7.15 ± 4.73	8.11 ± 4.23	14.07 ± 5.05	8.92 ± 5.32
12‐month follow‐up	6.59 ± 4.17	7.85 ± 4.89	15.15 ± 5.36	8.92 ± 5.67

*Note*: Higher scores indicate greater panic severity. Effect sizes for within‐group change from pretest to posttest were large (Cohen's *d* > 2.0 across groups).

The data met key parametric assumptions. PDSS scores were normally distributed at baseline and all follow‐ups (Shapiro–Wilk tests: *W* > 0.92, *p* > 0.11; skewness/kurtosis values between –0.82 and +0.71). Homogeneity of variance was confirmed via Levene's tests (*F* < 2.50, *p* > 0.09 at each time point). Box's *M* test indicated equality of covariance matrices across groups (F_M = 1.64, *p* = 0.036), supporting the validity of the mixed analysis of variance (ANOVA). Mauchly's test of sphericity was violated (*χ* ^2^ = 31.26, *p* < 0.001, *ε* = 0.76), so Greenhouse–Geisser corrections were applied to within‐subjects degrees of freedom. No outliers (>3 SD from group mean) were detected, and internal consistency of the PDSS remained high across assessments (*α* = 0.89–0.93).

The 3 (Group) × 4 (Time) mixed ANOVA revealed a significant main effect of Time (*F* = 94.18, *p* < 0.001, *η_p_* ^2^ = 0.60), indicating substantial overall reductions in panic severity across the study period. The main effect of Group was nonsignificant (*F* = 2.86, *p* = 0.065, *η_p_* ^2^ = 0.08), reflecting comparable baseline severity. Critically, the Time × Group interaction was highly significant (*F* = 7.65, *p* < 0.001, *η_p_* ^2^ = 0.20), demonstrating that symptom trajectories diverged over time (Table 2). This interaction, with a large effect size, underscores the differential long‐term impact of comorbid upper airway conditions on tDCS response.

**TABLE 2:** Mixed between‐within subjects ANOVA results for PDSS total scores.

Source	SS	*df* (G‐G corrected)	MS	*F*	*p*	*η_p_* ^2^
Time	23,608.19	2.28, 143.64	10,360.7	94.18	0.001	0.60
Time × Group	3906.69	4.56, 143.64	857.1	7.65	0.001	0.20
Group	9775.40	2, 63	4887.7	2.86	0.065	0.08

*Note*: df for between‐subjects factors are uncorrected.

Abbreviation: G‐G, Greenhouse–Geisser.

To decompose the significant Time × Group interaction, Bonferroni‐corrected (*α* = 0.05/3 = 0.017 per comparison) pairwise *t*‐tests were conducted on adjusted posttest residuals (covarying baseline PDSS scores). The results (Table 3) confirmed uniform acute response: all groups improved significantly from pretest to posttest (mean reductions: PD‐only = 12.81, PD + CRS = 12.50, PD + NSD = 9.15; all *t* > 15.2, *p* < 0.001, Cohen's *d* > 2.0). No significant between‐group differences emerged at posttest beyond a modest PD‐only versus PD + NSD gap (*p* = 0.018). At 6 months, all pairwise differences involving PD + NSD were significant (*p* < 0.005), reflecting early divergence. Critically, at 12 months, the PD‐only and PD + CRS groups remained indistinguishable (*p* = 0.998) and in sustained remission, while the PD + NSD group exhibited significant partial relapse relative to both (*p* < 0.001 and *p* = 0.022, respectively; mean increase from 6 to 12 months = 1.08, *t* = 2.58, *p* = 0.024). Overall, 85% of the PD‐only/PD + CRS participants achieved ≥40% sustained reduction (treatment response criterion), versus 54% in PD + NSD.

**TABLE 3:** Bonferroni‐corrected between‐group mean differences in adjusted PDSS scores at post‐treatment assessments.

Assessment	Comparison	Mean difference (95% CI)	SE	*t*	*p*
Posttest	PD‐only vs. PD + CRS	−0.53 (–3.61 to 2.55)	1.25	−0.42	1.000
	PD‐only vs. PD + NSD	−4.34 (–8.06 to –0.62)	1.51	−2.88	0.018
	PD + CRS vs. PD + NSD	−3.81 (–7.58 to –0.04)	1.53	−2.49	0.047
6‐month follow‐up	PD‐only vs. PD + CRS	−0.96 (–4.34 to 2.42)	1.35	−0.71	1.000
	PD‐only vs. PD + NSD	−6.92 (–10.89 to –2.95)	1.63	−4.24	0.001
	PD + CRS vs. PD + NSD	−5.96 (–9.99 to –1.93)	1.65	−3.61	0.002
12‐month follow‐up	PD‐only vs. PD + CRS	−1.26 (–4.73 to 2.21)	1.38	−0.91	0.998
	PD‐only vs. PD + NSD	−8.56 (–12.64 to –4.48)	1.67	−5.13	0.001
	PD + CRS vs. PD + NSD	−7.30 (–11.44 to –3.16)	1.69	−4.32	0.022

Abbreviations: CI, confidence interval; SE, standard error.

**FIGURE 1:**
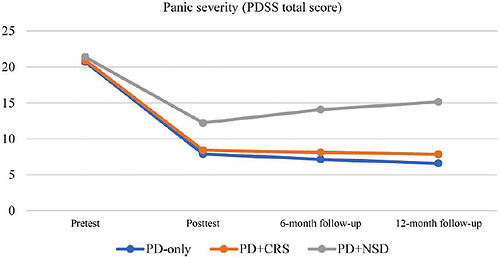
Changes in PDSS total scores over time by group.

As shown in Figure 1, all groups exhibited large and comparable reductions in panic severity immediately after the 25‐session tDCS protocol (57%–65% improvement). The PD‐only and PD + CRS trajectories remained flat and in the remission range (PDSS ≤ 8) throughout the 12‐month follow‐up. In striking contrast, the PD + NSD group displayed a clear upward trend after the 6‐month assessment, reaching moderate severity levels again by 12 months.

## 4 Discussion

The present study aimed to examine the efficacy and long‐term durability of anodal tDCS targeting the left dorsolateral prefrontal cortex (L‐DLPFC) in patients with panic disorder, with particular attention to the potential moderating role of comorbid upper airway conditions (chronic rhinosinusitis and nasal septum deviation). The current investigation sought to determine the efficacy and, critically, the long‐term stability of anodal tDCS targeting the L‐DLPFC in patients with PD stratified by the presence of upper airway comorbidities. The collective evidence yielded a compelling demonstration of the acute therapeutic power of the intervention, with all patient groups exhibiting immediate and substantial symptom reductions. This outcome aligns robustly with the prevalent neurobiological model of PD, which posits that the disorder involves functional deficits, particularly hypoactivity, in the L‐DLPFC—a key regulatory region responsible for exerting top‐down inhibitory control over subcortical fear circuitry, such as the amygdala and PAG (Sobanski and Wagner 2017). The application of anodal stimulation is hypothesized to restore or enhance this crucial cortical function, thereby diminishing the pathological activation of the central alarm system. This mechanism is consistent with numerous reports establishing tDCS as an effective neuromodulatory strategy for reducing anxiety symptoms across various affective disorders (Stein et al. 2020).

The most significant finding of this study was the pronounced divergence in long‐term symptom trajectory across the groups, which was captured by a highly significant Time × Group interaction. While the patients without upper airway issues and those with CRS maintained their substantial treatment gains over the entire 1‐year follow‐up period, demonstrating the robust and durable effect of L‐DLPFC tDCS in a substantial subset of patients, the group with NSD exhibited a distinct pattern of partial symptom relapse. This key finding indicates that a chronic mechanical obstruction to nasal airflow acts as a specific and powerful negative moderator that compromises the long‐term benefit of a centrally focused neuromodulatory treatment.

This differential outcome provides critical insight into the pathophysiology of PD. The untreated anatomical obstruction caused by NSD generates persistent interoceptive stress through mechanisms such as chronic sleep fragmentation and continuous subtle respiratory dysregulation (Alghamdi et al. 2022). According to the FSA theory, PD patients possess an intrinsically hypersensitive system for monitoring CO_2_ and O_2_ balance, which can be mistakenly triggered by minor physiological fluctuations, leading to panic (Preter and Klein 2008). The chronic, low‐level physical stress and altered breathing patterns inherent to NSD appear to continuously re‐sensitize this respiratory–panic pathway, effectively challenging and ultimately overwhelming the enhanced prefrontal regulatory capacity established by the tDCS intervention (Lee et al. 2021). The stability demonstrated in the PD + CRS group, whose comorbidity involves inflammation rather than fixed mechanical obstruction, supports this interpretation by suggesting that the mechanical restriction to breathing, rather than general systemic inflammatory burden, is the critical factor driving the long‐term instability. This mechanism is congruent with extensions of the FSA theory that propose a lifelong vulnerability in the suffocation alarm system, suggesting that persistent physical or psychological stressors can maintain or reactivate the pathological fear response (Preter and Klein 2008).

From a clinical perspective, these findings underscore the necessity of a holistic assessment for PD patients. The results suggest that the long‐term success of neurobiological interventions like tDCS is contingent upon addressing chronic peripheral stressors, especially those related to respiratory function. While tDCS is an effective acute treatment, comorbid, untreated NSD acts as a persistent negative moderator. Therefore, systematic screening and management of upper‐airway patency—potentially involving surgical correction for NSD—should be considered a prerequisite or concurrent therapeutic step to maximize the sustainability of remission achieved by L‐DLPFC tDCS. This integration of otolaryngological and neuropsychiatric care is essential to convert acute therapeutic success into enduring clinical recovery.

Several limitations warrant consideration. First, the quasi‐experimental design and purposive sampling limit causal inference and generalizability. Randomized controlled trials with larger, consecutively recruited samples are needed to confirm the present findings. Second, the NSD group was smaller (*n* = 13), although post hoc power for the critical Time × Group interaction exceeded 0.95. Third, objective physiological measures of nasal patency (e.g., acoustic rhinometry, peak nasal inspiratory flow) and nocturnal oximetry were not obtained longitudinally; future studies should incorporate these to directly test the proposed interoceptive mechanism. Fourth, although medication regimens for panic disorder were kept stable, other unmeasured factors—such as lifestyle modifications, increased physical activity, improved sleep hygiene, or natural fluctuations in symptom severity—could have contributed to the observed reductions in panic symptoms or attack frequency across groups. Finally, concomitant medication use—while stable—was not fully quantified; subtle dose adjustments below clinical detection thresholds cannot be entirely ruled out. Despite these limitations, the 1‐year follow‐up, gold‐standard outcome measure (PDSS) and clear differentiation between inflammatory (CRS) and obstructive (NSD) airway pathology provide robust preliminary evidence that chronic mechanical nasal obstruction is a treatable moderator of long‐term outcome in neuromodulation for panic disorder.

## 5 Conclusion

Anodal tDCS targeting the left DLPFC is a highly effective, safe, and non‐pharmacological treatment for panic disorder, yielding rapid remission in most patients and durable benefit through 1 year when structural nasal obstruction is absent. Untreated nasal septum deviation, however, functions as a powerful negative moderator, precipitating partial relapse through chronic re‐sensitization of respiratory–panic pathways. These findings mandate routine upper‐airway screening in PD and strongly advocate an integrated neuropsychiatric–otolaryngological treatment model: combining L‐DLPFC tDCS with targeted correction of nasal airflow offers the greatest likelihood of converting acute response into lifelong recovery.

## Author Contributions

**Zahra Alipour**: software, formal analysis, resources. **Ahmad Alipour**: validation, visualization, supervision. **Roghayeh Mohammadi**: conceptualization, methodology, data curation, investigation, funding acquisition, writing – original draft, writing – review and editing, project administration.

## Funding

The authors have nothing to report.

## Ethical Considerations

The study was approved prospectively by the Ethical Committee of Payame Noor University. All participants provided written informed consent before participation.

## Conflicts of Interest

The authors declare no conflict of interest.

## Data Availability

The datasets generated during and/or analyzed during the current study are available from the corresponding author on reasonable request.
